# Advancing the conversation: next steps for lesbian, gay, bisexual, trans, and queer (LGBTQ) health sciences librarianship

**DOI:** 10.5195/jmla.2017.206

**Published:** 2017-10-01

**Authors:** Blake W. Hawkins, Martin Morris, Tony Nguyen, John Siegel, Emily Vardell

## Abstract

In recent years, librarians in various sectors have been moving forward a conversation on the distinct information needs and information-seeking behavior of our lesbian, gay, bisexual, trans, and queer (LGBTQ) patrons and how well the profession recognizes and meets those needs. Health sciences librarianship has been slower than other areas of the profession in creating an evidence base covering the needs of its LGBTQ patrons, with, until recently, only very limited literature on this subject. LGBTQ health sciences librarianship is now starting to attract new interest, with librarians working together to bring this emerging specialization to the attention of the broader professional community. In this paper, the authors report on a dedicated panel discussion that took place at the 2016 joint annual meeting of the Medical Library Association and Canadian Health Libraries Association/Association des bibliothèques de la santé du Canada in Toronto, Ontario, Canada; discuss subsequent reflections; and highlight the emerging role for health sciences librarians in providing culturally competent services to the LGBTQ population. Recommendations are also provided for establishing a tool kit for LGBTQ health sciences librarianship from which librarians can draw. We conclude by highlighting the importance of critically reflective practice in health sciences librarianship in the context of LGBTQ health information.

## INTRODUCTION

In recent years, librarians in various sectors have been moving forward a conversation on the distinct information needs and information-seeking behavior of our lesbian, gay, bisexual, trans, and queer (LGBTQ) patrons and how well the profession recognizes and meets those needs. Health sciences librarianship has been slower than other areas of the profession [1, 2] in creating an evidence base covering the needs of its LGBTQ patrons: until 2016, only three articles reported on this subject [3–5]. However, the field is now starting to attract new interest, with librarians working together to bring issues around LGBTQ health sciences librarianship to the attention of the broader professional community [6–8].

To develop this conversation, two of the authors of the present article (Hawkins, Morris) convened a panel of interested librarians at the 2016 joint annual meeting of the Medical Library Association (MLA) and Canadian Health Libraries Association/Association des bibliothèques de la santé du Canada (CHLA/ABSC) to consider how health sciences librarianship should best respond to the distinct and evolving needs of our LGBTQ patrons and how the profession might drive forward a research agenda to increase the evidence base in this area. To gain a broad perspective, we made a deliberate attempt to include panel members from a variety of backgrounds, such as traditional health sciences librarians based at universities or hospitals, researchers, and a nonlibrarian panelist, Ryan Dyck, who had specific expertise in information-seeking behavior and a considerable background in community-based LGBTQ health services. A brief biography of each panelist is provided in the supplemental appendix to this article.

Dyck opened the session with an introduction to everyday challenges that LGBTQ people face regarding their health, clarifying that it is not very difficult to build networks between LGBTQ patrons and the library. He also introduced various background resources for LGBTQ health, such as Fenway Health reports [9]. Each panelist then gave a short presentation, covering current issues in LGBTQ health information, the information needs of LGBTQ health sciences library patrons, and how health sciences librarians can develop and improve their practice in this context. We concluded with an audience discussion that further explored these themes, specifically covering sources of information and ways of getting engaged as a non-LGBTQ person.

The session attracted significant social media interest (particularly on Twitter [10]), and the months following it have provided an opportunity for the panelists to reflect on ways to develop and improve the relationship between health sciences librarianship and its LGBTQ patrons. This paper provides a summary of the panelists’ conversations that took place during the session and concludes with a discussion of our subsequent reflections and with some recommendations that flow from them. In sharing our thoughts in this paper, we invite colleagues to reflect on this aspect of their own practice and to consider joining the scholarly discussion now taking place.

## LESBIAN, GAY, BISEXUAL, TRANS, AND QUEER (LGBTQ) CULTURAL COMPETENCY TO IMPROVE HEALTH INFORMATION SERVICES

Tony Nguyen, AHIP, is involved in developing LGBTQ cultural competency training for health sciences librarians [11]. He opened the panel discussion with an outline of how librarians can improve their cultural competency around LGBTQ people and why they should consider doing so, drawing attention to the many ways that exist to approach the diverse populations in the United States and beyond.

**Figure 1:**
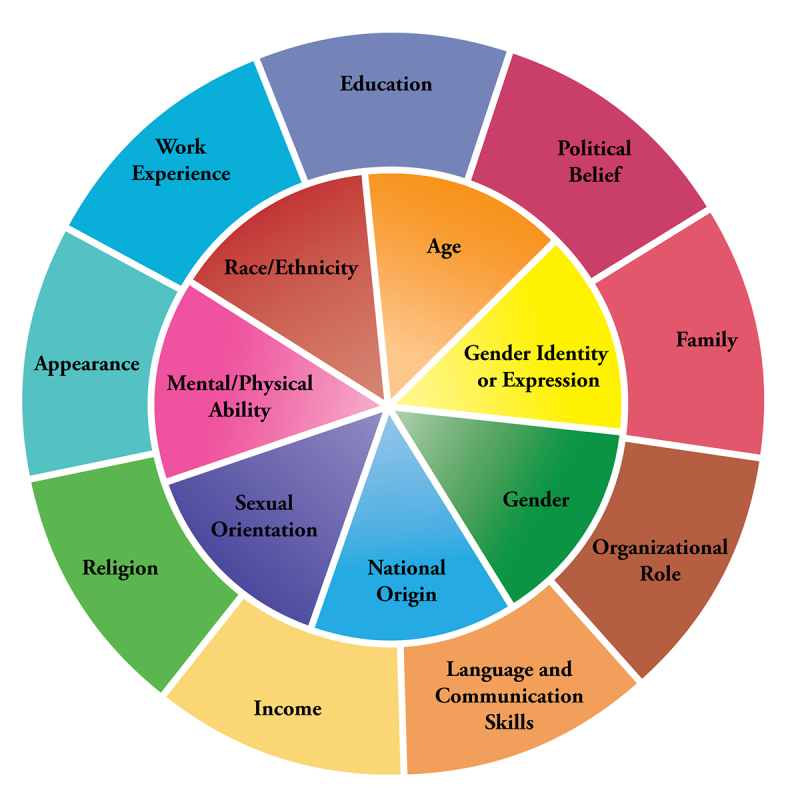
Diversity Wheel as used at Johns Hopkins University [12]

Before delving into LGBTQ-specific cultural competencies, it is important to explore diversity and cultural competencies in general. One effective way to facilitate such exploration is the “Diversity Wheel,” now being used by many different diversity and inclusion offices (Figure 1); Johns Hopkins University of Medicine’s Diversity Leadership Council shares a model that is available to view online [12]. According to the Johns Hopkins Diversity Leadership Council, the outer portions of the Diversity Wheel represent dimensions of oneself that commonly change over time such as income or work experience, while the inner portions of the wheel represent internal dimensions that are frequently more permanent or visible by members of the community, which include race and sexual orientation.

Some care should be taken with this distinction, however. Meyer and McHugh argue that gender identity, for example, is a social and psychological construct with little scientific evidence that it is an innate, biological property [13]. Furthermore, some individuals, such as genderqueer people, may not identify with any gender or may identify with a combination of male and female genders. For such people, gender identity is likely to be a far more fluid characteristic than the wheel might suggest. Nevertheless, while the inner dimensions of the diversity wheel should be considered carefully, the wheel can be a helpful tool to begin discussions on cultural competency.

The combination of each section of the diversity wheel influences how individuals act, their belief systems, and their values, experiences, and expectations [12]. Purnell demonstrates that these different facets do not just contribute to one’s unique individuality, but also reflect the community in which a person lives and society at large [14]. If one fills out the wheel with one’s personal information, one can begin to learn and understand the differences between and commonalities with colleagues and those to whom one provides service or, in terms of health care, the patients one treats.

When investigating LGBTQ cultural competency, a better understanding of the unique differences of three categories—sex, gender identity, and sexuality—can improve the profession’s support of the community. “Sex” refers to one’s genetic and anatomic identity. An individual can be born with XX or XY chromosomes (or indeed a different combination of chromosomes [15]). It is important to note that an individual’s genitalia might not match the chromosomes they are assigned at birth. A famous example would be David Reimer, born in the 1960s as a twin. Though male, he was raised as a female due to a botched circumcision. John Money, a psychologist at Johns Hopkins Hospital, studied both twins to determine whether gender identity is created socially or is biological. Reimer suffered from severe depression for years due to being raised a female and believing himself to be male. Upon learning of the gender reassignment, Reimer later committed suicide [16].

“Gender identity” refers to the personal perception that an individual has of their gender; in other words, does one identify as a man, a woman, or an other identity? Gender identity is thus an internal and central part of one’s sense of self. This can be contrasted with a person’s gender role, or “gender expression,” which is how a person outwardly chooses to appear based on their actual or perceived sex. Gender expression is the external expression of one’s gender identity.

Finally, “Sexuality” refers to which gender a person is sexually or romantically attracted. People can be attracted to the same gender (gay/lesbian), both genders (bisexual), or the opposite (heterosexual) gender, or they may also have no sexual or romantic attraction to any gender. However, sexuality is more complex. For example, a person may identify as heterosexual; however, their sexual behavior could be that they are sexually active with individuals of both sexes, the opposite, or the same sex. Knowing and understanding the complexities of sex, gender, and sexuality can give health sciences librarians the opportunity to be a bridge between LGBTQ populations and health care providers.

**Figure 2:**
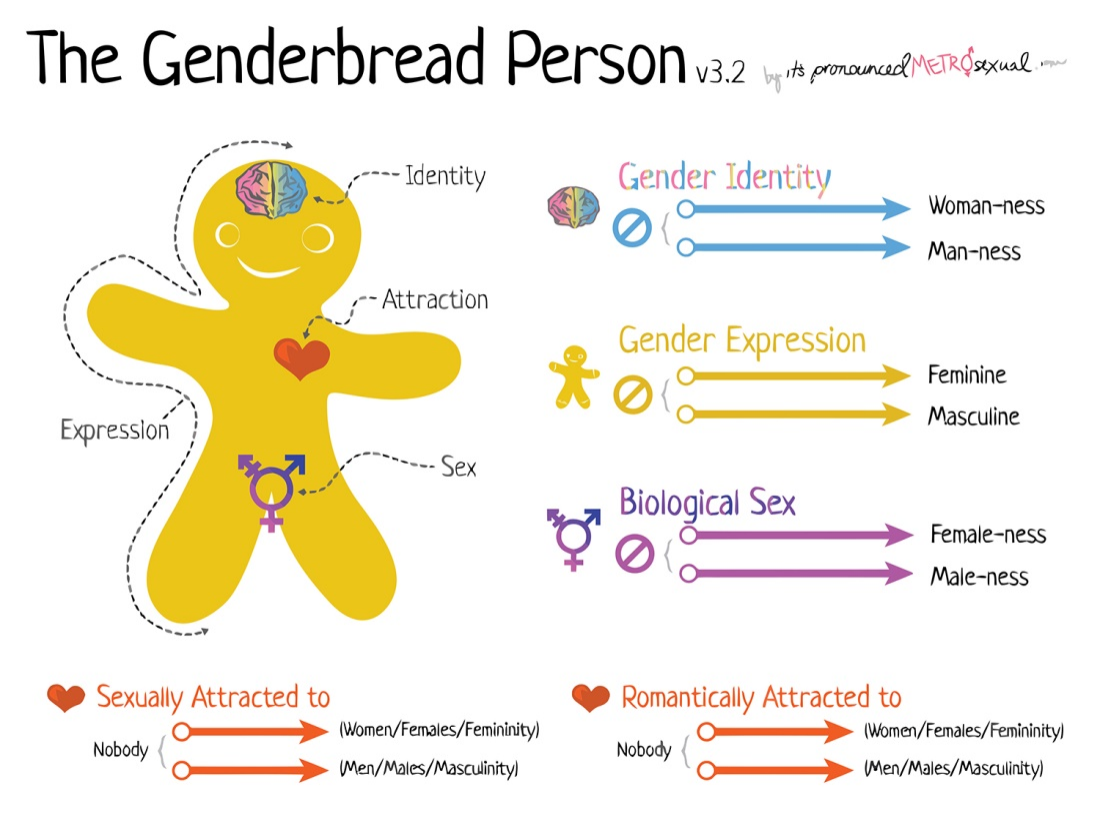
The Genderbread Person [17]

Once one understands the complexities of sex, gender identity, and sexuality, then one can begin to comprehend many of the health issues that affect the LGBTQ population and the social determinants and factors that influence them. The “Genderbread Person” is a graphic visualization that tries to address these complexities [17], albeit simplistically, and we recommend it as an accessible introduction into sex, gender, and sexuality. Importantly, it demonstrates how these three aspects of a person are not binary but, in fact, are part of a spectrum. In Killermann’s version of the Genderbread Person (Figure 2), a cookie cutout of a person with images and arrows is employed to highlight the identity, expression, sex, and attraction of the person. To the right of the images are spectrum details of gender identity, gender expression, biological sex, and romantic and sexual attraction [17].

However, sex, gender, and sexuality are much more complicated than the Genderbread Person suggests. For example, the chart’s section on gender expression is focused on feminine and masculine roles but does not address those who identify as third gender (i.e., a person who does not characterize theirself as either male or female). It also ignores the effect of cisnormativity (i.e., the standard of normalcy in a culture that tends to privilege individuals whose sense of personal identity and gender corresponds with their birth sex) and gender, for example. Despite these drawbacks, the Genderbread Person, like the Diversity Wheel, can be a useful starting point for constructive discussions.

Becoming familiar and comfortable with the terminology that the LGBTQ population uses in identifying and describing themselves will also lead to improved services and support for this population. This can improve the cultural sensitivity awareness that librarians can have in welcoming this population and supporting their information needs.

## HEALTH LIBRARIANS AND LGBTQ YOUTH

Blake Hawkins continued the session with reflections on the health information behaviors and needs of LGBTQ youth drawn from his own research in this area [18, 19] and discussed specific considerations that health sciences librarians should remember when interacting with this group. He emphasized that LGBTQ youth have a variety of lived experiences, that these experiences can influence how members of this group seek health information, and that these ways of seeking information may differ from those of older LGBTQ people. It is important for health librarians to recognize these varying experiences and be open minded when building relationships with potential library users, such as with community organizations or campus queer–straight alliances.

His discussion took as a starting point the 2010 *It Gets Better* campaign, which in a problematic fashion attempted to provide motivation for LGBTQ youth [20, 21]. A person unfamiliar with the LGBTQ community might think all LGBTQ youth are defined by negative lived experiences and by a lack of positive opportunities. The mainstream media, with its tendency to provide a “one-size-fits-all” homogenous approach to describe the everyday lives of LGBTQ youth, frequently focuses on the high rates of depression, self-harm, and suicide that this group has experienced [22], thus perpetuating this negative perception. The *It Gets Better* campaign, albeit not a campaign of the mainstream media, did not challenge these preconceived, negative ideas about the everyday lives of LGBTQ people. Furthermore, despite being intended to be a form of mental health promotion, the campaign lacked helpful advice on seeking relevant health information and missed an opportunity to discuss the lack of reliable sources of health information for this group.

Online channels are a popular place to find health information, which is particularly true for LGBTQ youth due to a lack of more reliable information sources and the opportunity for LGBTQ youth to ask questions about their health or different conditions without fear of stigma [23, 24]. However, to the best of the panel’s knowledge, these spaces do not have any health care or health sciences librarians acting as moderators, meaning that they have the potential of providing inaccurate information. For example, in one study that Hawkins co-conducted in the online *Gay Teen Forum* [25]*,* participants erroneously stated that HIV transmission between partners practicing unprotected sex in a monogamous relationship becomes impossible after one week, whereas, in fact, this can take over a month [26]. Based on member comments, some of the youth believed this post and potentially followed this advice in their health practices. Participation in such forums as a moderator or trusted information source can potentially be a good opportunity for health sciences librarians, particularly consumer health librarians.

Hawkins’ other work with LGBTQ youth in British Columbia demonstrates that the lived experiences of LGBTQ youths who come to a librarian for health information are likely to affect their information-seeking behavior [18]. His findings are highly consistent with those of other panelists [6, 27]: that many members of this group fear stigma and need to know that the librarian will demonstrate cultural competency when handling their information requests. They also would like the librarian to take the time to build relationships. One effective way for librarians, particularly consumer health librarians, to gain a better understanding of the needs of their LGBTQ patrons is to participate in safe space training (i.e., a way for those who are supportive of LGBTQ people in an institution to identify themselves publicly as well as receive training on terminology and support for LGBTQ colleagues) or to build relationships by attending meetings of relevant community organizations, ideally on a regular basis. Such activities and relationship building take time, empathy, and a willingness to understand the distinct needs of LGBTQ youth. However, if the librarian is working in a setting where this kind of relationship building is possible, it would be particularly welcomed by this user group and can result in long-term connections as well as services that better serve the needs of this user group.

Opportunities, therefore, exist for interested health sciences librarians in a variety of settings to gain a better understanding of the context and information needs of LGBTQ youth. In fact, the strategies suggested here could be employed by librarians in all sectors and not just health. Consumer health librarians could work with relevant community organizations or consider the possibility of providing information through online forums, while academic librarians could attend meetings of their campus queer–straight alliances. Librarians also have a role to play in improving the availability of targeted health information to this group by, for example, focusing on LGBTQ youth when researching and creating reliable information.

## LGBTQ CONSUMER HEALTH INFORMATION

Emily Vardell continued the session by providing insights gained from her doctoral work on LGBTQ consumer health information. Appropriate consumer health information has not always been available for the LGBTQ community. This unmet information need was in starkest relief in the United States during the height of the HIV/AIDS crisis. Evidence-based information about HIV/AIDS was in short supply, and the amount of misinformation circulating might have seemed insurmountable at the time. Distrust of consumer health information has been a continuing issue for the LGBTQ community. Medical librarians can assist with providing quality consumer health information and combating misinformation with many different populations, including the LGBTQ community. Being visible as a safe and supportive space can begin with having LGBTQ consumer health information available at a library in the form of printed pamphlets, LibGuides, and displays.

One specific area in which librarians can connect LGBTQ patrons with needed information is by locating LGBTQ-friendly providers and health care facilities. The Gay and Lesbian Medical Association has a Provider Directory that is searchable by zip code, state, and/or community partner [28]. The Human Rights Campaign publishes an annual Healthcare Equality Index that provides information and metrics on health care facilities’ policies and practices relating to LGBTQ patients, visitors, and employees [29]. The 2017 edition reported that a record 99% of responding institutions included both sexual orientation and gender identity in their patient nondiscrimination policies, but that unfortunately only 39% had policies that “specifically outline procedures and practices aimed at eliminating bias and insensitivity, and ensuring appropriate, welcoming interactions with transgender patients” [29].

A unique aspect of LGBTQ consumer health is that it may quickly overlap with other information areas including law and support systems. Organizations that work in this area include the National Center for Lesbian Rights, National Center for Transgender Equality, Transgender Law, and Parents and Friends of Lesbians and Gays. An example of the overlap between LGBTQ consumer health and law is the implications of the United States Affordable Care Act for the LGBTQ community. The Affordable Care Act (as it stood at the time of publication) prohibits refusing coverage due to preexisting conditions, which is a significant advancement for those with HIV/AIDS as well as transgender individuals. Under the Affordable Care Act, there are no more lifetime dollar limits, which means increased coverage for long-term, comprehensive treatment of chronic diseases and the costs of gender confirmation surgeries (in plans that cover this).

It is important for librarians to understand that just as health care providers in general have different information needs from those receiving care, it is also true that providers of health care to LGBTQ people have specific information needs that are distinct from the information needs of LGBTQ patients. To that end, librarians can support both LGBTQ health care professionals and the LGBTQ recipients of that care through providing appropriate and culturally competent reference services and by facilitating access to targeted information. For example, librarians can create LibGuides that curate resources for health care providers who treat LGBTQ populations, which could include resources for appropriate terminology. Librarians can also assist with advocating for the inclusion of LGBTQ issues in the curriculum for health care professions (e.g., medical, nursing, pharmacy, public health). There is also a role for librarians in assisting health care providers by developing robust database search strategies that encompass LGBTQ issues, which often require sophisticated use of controlled vocabulary and synonyms.

A significant aspect of working with the LGBTQ community is being sensitive to the importance of using appropriate vocabulary. While the best practice may indeed be asking the individual what their preferred pronouns and other terms are (e.g., gay, lesbian, trans), resources are also available for obtaining terms and definitions specific to gender identity. The “Top Health Issues for LGBT Populations Information & Resource Kit” from the Substance Abuse and Mental Health Services Administration is a particularly helpful guide for navigating community-specific language [30].

Medical librarians are well positioned to serve as information advocates for access to quality consumer health information and combat misinformation about and for the LGBTQ community.

## LIBRARIAN CONFIDENCE IN MEETING LGBTQ INFORMATION NEEDS

John Siegel, AHIP, is conducting research into the ability and willingness of academic librarians to serve LGBTQ patrons. As noted by other speakers, as attention to and awareness of LGBTQ issues increases, librarians should be equipped to respond this increase and to assist health and medical researchers and patrons with LGBTQ-themed inquiries. A number of studies were conducted with physicians, mental health counselors, and other health professionals regarding LGBTQ knowledge and training needs, such as those by Lapinski, Sexton, and Baker [31] and Oswalt, Evans, and Drott [32]. However, there is a lack of LGBTQ research in this area pertaining to librarians and, as already noted, close to none relating to health sciences libraries and librarians. This prompted Siegel’s current study to determine librarians’ knowledge of LGBTQ information resources and the need for additional training.

Siegel designed a 20-question survey incorporating both quantitative and qualitative questions to determine the skill and comfort level of librarians responding to sexual orientation and gender identity information inquiries. Participants were recruited through electronic discussion lists focused on health librarianship such as MEDLIB-L (MLA) and HSIG-L (Association of College and Research Libraries Health Sciences Interest Group) and those serving broader audiences in academic, public, state, and special libraries, including ULS-L (University Libraries of the American Library Association), PUBLIB (public librarianship), ARSL-L (Association for Rural and Small Libraries), and ARKLIB-L (Arkansas Library Association). There were a total of 1,410 survey respondents, of which most (72%) were librarians. The remaining respondents were paraprofessionals (15%), were students in library and information sciences (8%), or functioned in other capacities (5%) such as library science teaching faculty. Siegel is currently focused on an analysis of librarian responses.

In addition to demographics, the survey asked about familiarity with 15 LGBTQ-related terms. Most librarian respondents (90% or higher) were familiar or very familiar with more commonly used terms such as sexual orientation, gay, lesbian, bisexual, and trans. Approximately half of these respondents were less familiar with or had never heard of gender identity terminology such as genderqueer, cisgender, gender binary, and gender variant. The American Psychological Association points out that language concerning sexual orientation and gender identity is constantly evolving [33]. This factor illustrates the continued need for professional development.

The majority (80%) of librarian respondents agreed or strongly agreed that they would benefit from additional training to help serve LGBTQ information needs. Open-ended comments revealed a lack of ability to respond to LGBT-themed inquiries. Common themes included the need to learn about current information resources, participate in instruction on answering questions related to sexuality (including sexual orientation), and gain practical advice for making their libraries welcoming to the LGBT community.

Interestingly, librarian respondents to the survey raised larger issues that presented barriers to responding to LGBTQ information needs, such as community or institutional culture. One respondent indicated in an open-ended comment that the “[c]ommunity lashes back with negative comment cards for book displays about LGBTQ pride month.” Several librarians indicated concerns about collection development. In one respondent’s library “[LGBTQ] materials tend to circulate less, so they get weeded [removed from the collection].” Also, the Library of Congress subject headings were mentioned as “terrible” for queer and transgender-related topics, which limits the discoverability of resources in library online catalogs. Another respondent indicated challenges with updating integrated library systems to reflect preferred names for transgender patrons. Besides receiving training, it is essential for librarians to evaluate and modify library policies, procedures, and systems as needed to ensure that LGBTQ information needs are being met.

Given the interest in training that survey respondents expressed, Siegel is in the process of planning an all-day pre-conference as part of an upcoming Arkansas Library Association conference. If the pilot is successful, he hopes to refine the LGBTQ training and collaborate with other libraries to develop a standardized program that can be adopted by a variety of libraries.

## DISCUSSION

Co-moderator Martin Morris drew together the themes of the panel, incorporating his own reflections, and chaired the audience discussion. He noted that the LGBTQ community is an underserved population in the context of librarianship as a whole [34] and that the conversation around LGBTQ health librarianship, which the panelists seek to advance, connects to and can be informed by broader conversations within librarianship. He added that many LGBTQ patrons do not believe that medical librarians are able or willing to meet their information needs [3, 6], a situation that requires attention in the profession.

In *The Atlas of New Librarianship*, Clark lists three fields where librarians who support traditionally underserved or marginalized communities can choose to focus their efforts: reference, outreach, and research [35]. Two additional fields are also relevant: cataloging [36–38] and collection management [39]. The panel demonstrated that while there is less published research in health sciences librarianship on responses to developing needs of LGBTQ patrons, individual librarians are conducting unpublished work that covers all five of these foci. This demonstrates both that there is a need for health sciences libraries to modify their practice and that there are sources of expertise and models from which to draw.

We believe that the next step is to build on the work of individual practitioners to ensure that all health sciences librarians have the tools and resources to meet the needs of members of the LGBTQ community who come to librarians for assistance or who feel prevented from doing so. Achieving this will involve two steps: (1) build and implement a tool kit of interventions that attract LGBTQ users to libraries and (2) encourage critically reflective thinking in health sciences librarianship. Understanding why interventions to support LGBTQ patrons have emerged will foster new strategies to promote cultural competency in health sciences librarianship and better support the LGBTQ population, as well as other underserved groups.

### Building and implementation of a tool kit for LGBTQ health librarianship

There is now significant and consistent evidence demonstrating that many LGBTQ prospective patrons do not request the assistance of medical librarians, either because they believe that librarians are unequipped to meet their needs or through fear of discrimination. In general, LGBTQ patrons strongly prefer to work with health sciences librarians who also identify as LGBTQ [3, 6].

Respondents to those studies consistently suggest various strategies that library workers can adopt to proactively show that they welcome and are able to handle LGBTQ-specific questions [3, 6]. Suggestions include participation in Safe Space training, display of visible signs of support, appropriate collection development, creation of LGBTQ-specific resources such as dedicated subject guides or resource web pages, and involvement with appropriate organizations such as youth groups. Additional options are creating a distinct subject area for LGBTQ health that would be assigned to an interested (and if necessary trained) librarian and using cultural and linguistic variances in library instruction and reference services. Different strategies may be appropriate depending on whether the library is in a consumer health or academic setting.

We would like to see the development of an accessible evidence base or tool kit that librarians could draw from when working to make their libraries more accessible and welcoming to potential LGBTQ patrons. The development of such a tool kit would call on librarians to implement and evaluate these and other possible strategies in their institutions and make the results publicly accessible, for example, through publication as journal articles. Such “tried-and-tested” work could also form part of dedicated training for librarians. There would also be a role for representative organizations such as MLA and CHLA/ABSC in making colleagues aware of this research and training, for example, through dedicated web pages.

There are also strategies to be adopted at a national and international level, such as addressing outdated, discriminatory, or inadequate Medical Subject Headings (MeSH). The above strategies, once considered and implemented in an institution, are likely to improve the patronage of LGBTQ users, and we would welcome research into their implementation and effectiveness.

### Critically reflective health sciences librarianship

From studies such as that by Schroeder and Hollister [40], we know that many librarians are committed to social justice issues and are willing to make changes to their practice to address these issues. Siegel’s work demonstrates that most health sciences librarians are able to handle questions related to sex and sexuality or are held back purely by a lack of knowledge rather than by discomfort with the subject matter and would welcome training in this area [27]. Considering this, it is interesting to speculate why more health sciences libraries do not adopt proactive measures to address the concerns of LGBTQ patrons. It is possible that librarians believe these measures are not necessary due to significant improvements in civil rights for LGBTQ people in recent years (despite the reality that this is drastically less true for bisexual and trans people than for lesbians and gay men, for example) or because they presume there are no significant differences between LGBTQ and non-LGBTQ health. Alternatively, they may believe that adopting such measures would be contrary to the spirit of a neutral library service, where all are inquiries are treated equally and objectively.

In response, we encourage the greater use of critical reflective practice in health sciences librarianship. This practice, in its simplest form, is a way for professionals to reflect on their work in a structured way and is particularly useful when one is considering areas of practice that may be unfamiliar and deciding what to do differently next time [41, 42]. Grant notes that health sciences librarians have not been particularly avant-garde in adopting this practice, despite their proximity to evidence-based medicine [43]. We advocate employing critical reflection as a technique—for example, by using the Diversity Wheel [12]—to consider how dimensions of one’s personality and background such as sexuality or gender shape one’s approach to working with those who are different than oneself. While it is important to remember that LGBTQ people are not homogenous in their lived experiences, a reflective understanding of the effect of differences in lived experience between oneself and others may help health sciences librarians go outside their comfort zones or persuade them of the necessity for targeted interventions when considering how best to provide equitable support to LGBTQ people.

The increasing recognition of the necessity for librarians to undertake such critically reflective thinking into the way they serve marginalized or less privileged patrons has led to the rise of the critical librarianship movement [44]. Critical librarianship is a “movement of library workers dedicated to bringing social justice principles into our work in libraries” [45] and offers a way for librarians to critique how they serve populations that are different from themselves. An accessible introduction to critical librarianship is provided by Clark [46]. It is a source of support and mentorship for librarians who wish to critically reflect on their professional practice and potentially change it based on the results of that reflection. In addition to encouraging critical self-reflection, critical librarianship also questions the idea of library neutrality [47], noting that a library that aims to be strictly neutral (an aim that, incidentally, few if any libraries achieve) risks demonstrating indifference to marginalized populations, such as LGBTQ people. However, while it has thought-provoking implications for health sciences librarianship and is, at its core, uncomplicated, critical librarianship has also been criticized as being “inaccessible, exclusionary, elitist, and disconnected from the practice of librarianship” [48], which may partially explain its limited impact to date. Despite these criticisms, we believe that critical librarianship raises various issues that are relevant to health librarianship and can contribute to the critically reflective thinking we recommend to colleagues who are considering how to modify their practice.

## CONCLUSION

Our purpose in conducting the panel discussion and in writing this article based on its content and our subsequent reflections has been to advance the developing conversation on LGBTQ health sciences librarianship, encourage colleagues to engage with this discussion, and place initiatives currently developed and implemented by the health librarianship community in the scholarly record. Our reflections suggest that this emerging field includes several distinct facets that require further development. These areas include publication of an evidence base of modifications to our practice, research into the effectiveness of such interventions, and the increased use of critical reflective practice in health sciences librarianship.

The distinct needs of LGBTQ people are becoming more prominent as societal attitudes develop, and health sciences librarians have a role to play to ensure that the information needs of this community are met. The authors are not aware of any statement by a health library organization specifically covering diversity and equity or that lays out a path for the development of these issues in the profession. We do note, with interest, that MLA is taking steps in this direction by developing related updated strategic goals [49] and by significantly raising the profile of diversity and equity issues in its updated outline of professional competencies. These updated competencies call on health sciences librarians to integrate appreciation of diversity and equality into professional practice, to be able to describe one’s own cultural background and recognize bias, and to develop and implement practices that foster diversity and quality [50]. We look forward to these updated strategic goals and professional competencies and welcome the increased focus on diversity issues that they bring to health sciences librarianship. We also invite our colleagues—whether in an academic, hospital, consumer health, or other setting—to consider how they can contribute to the work of developing our service to LGBTQ patrons and to draw on the developing sources of expertise and good practice available.

## SUPPLEMENTAL FILE

Appendix Biographies of participants in the “Creating a Needed Dialogue: A Discussion About Lesbian, Gay, Bisexual, Transgender, Questioning (LGBTQ) Health Librarianship in 2016” session during Mosaic ’16
