# Correction

**DOI:** 10.5195/jmla.2018.410

**Published:** 2018-01-02

**Volume 105**

**105(4) October, page 325**

Hawkins BW, Morris M, Nguyen T, Siegel J, Vardell E. Advancing the conversation: next steps for lesbian, gay, bisexual, trans, and queer (LGBTQ) health sciences librarianship. J Med Libr Assoc. 2017 Oct;105(4):316–27. DOI: http://dx.doi.org/10.5195/jmla.2017.206.

The first author name in reference #11 is incorrect. The reference should be:

11. Leskovec J, Nguyen T. Improving the health, safety and well-being of LGBT populations [Internet]. Baltimore, MD: National Network of Libraries of Medicine; 2017 [cited 20 Feb 2017]. <https://nnlm.gov/classes/LGBTHealth>.

