# Supplementary material for: Advancing the conversation: next steps for lesbian, gay, bisexual, trans, and queer (LGBTQ) health sciences librarianship
Source: J Med Libr Assoc. 2017 Oct 1;105(4):316–27. doi: 10.5195/jmla.2017.206 (PMC5624421; doi:10.5195/jmla.2017.206)
Supplement: Appendix [file jmla-105-316-s001.pdf]

JMLA Journal of the Medical Library Association

Supplemental content to
J Med Libr Assoc. Oct;105(4): dx.doi.org/10.5195/jmla.2017.206
www.jmla.mlanet.org
© Hawkins, Morris, Nguyen, Siegel, Vardell 2017

Advancing the conversation: next steps for lesbian, gay, bisexual, trans, and queer (LGBTQ) health sciences librarianship

Blake W. Hawkins, MLIS Student; Martin Morris, MSc; Tony Nguyen, MLIS, AHIP; John Siegel, MLS, AHIP; Emily Vardell, PhD, MLS

APPENDIX

Biographies of participants in the "Creating a Needed Dialogue: A Discussion About Lesbian, Gay, Bisexual, Transgender, Questioning (LGBTQ) Health Librarianship in 2016" session during Mosaic '16

Ryan Dyck was, at the time of the panel, director of Egale's Research, Policy and Development Department, which is responsible for compiling, producing, and communicating the best available evidence, information, and tools for promoting and fostering the human rights and equitable inclusion of lesbian, gay, bisexual, trans, and queer (LGBTQ) people throughout all aspects of society.

Blake Hawkins is a graduate student at the University of British Columbia, who recently completed his master's thesis, *Let's Map It Out: The Everyday Health Information Seeking of LGBTQ Youth in Prince George, British Columbia.* He is interested in the information behaviors of underserved communities, health policy development, human-computer interactions, knowledge syntheses methods, and creative and participatory research methods.

Martin Morris is a health sciences liaison librarian at McGill University, Montreal, Quebec, Canada. He has previously worked as a hospital librarian in Montreal and as a public librarian in the United Kingdom. His interests include providing library and information services to LGBTQ people, knowledge synthesis methodologies, and the spread of innovations in library and information settings.

Tony Nguyen, AHIP, is the technology and communications coordinator at the National Network of Libraries of Medicine, Southeastern/Atlantic Region. He assesses and identifies needs to develop programs to support the needs and interests of unaffiliated, inner city, and minority health professionals; the public health workforce; public and health sciences librarians; community groups; and health consumers.

John Siegel, AHIP, was, at the time of the panel, student success librarian and associate professor at the University of Arkansas at Little Rock. In addition to providing support for first-year students in general education courses, he served as liaison for health sciences, biology, psychology, and rehabilitation counseling. Previously, Siegel was reference librarian at the University of Pittsburgh's Health Sciences Library System.

Emily Vardell was, at the time of the panel, a doctoral candidate and teaching fellow at the School of Information and Library Science at the University of North Carolina at Chapel Hill. Her research focused on health information behavior, particularly health insurance literacy and decision making.
